# Estimating the carbon footprint of household activities in Japan from the time-use perspective

**DOI:** 10.1007/s11356-022-23387-w

**Published:** 2022-10-26

**Authors:** Yida Jiang, Ryoko Motose, Tomohiko Ihara

**Affiliations:** 1 Graduate Program in Sustainability Science - Global Leadership Initiative, The University of Tokyo, 5-1-5 Kashiwanoha, Kashiwa, Chiba, 277-8563 Japan; 2 Research Institute of Science for Safety and Sustainability, National Institute of Advanced Industrial Science and Technology, 16-1 Onogawa, Tsukuba, Ibaraki, 305-8569 Japan; 3 Department of Environment Systems, Graduate School of Frontier Sciences, The University of Tokyo, 5-1-5 Kashiwanoha, Kashiwa, Chiba, 277-8563 Japan

**Keywords:** Carbon footprint, Time-use perspective, Household activities, Consumption behavior, Carbon mitigation, Sustainable lifestyle

## Abstract

The household sector is a major driver of energy consumption and greenhouse gas (GHG) emissions. However, most existing studies have only estimated households’ carbon footprint from their expenditures. Households’ daily activity time, a scarce resource that limits and determines their consumption behavior, has rarely been integrated into the estimation. Incorporating the daily time-use patterns should thus provide a more practical perspective for mitigation policies aiming at promoting sustainable household lifestyles. In this study, by linking household time-use data and expenditure data of Japan, the carbon footprint and the GHG intensity of time of 85 daily household activities constituting the 24 hours in a day are estimated. Compared to the maximal 20-activity disaggregation in existing studies, our detailed 85-category disaggregation of daily time enables unprecedented details on the discrepancies between the carbon footprint from daily activities, many of which have previous been treated as one activity. Results indicate significant carbon mitigation potential in activities with a high GHG intensity of time, such as cooking, bathing, and mobility-related and activities. Average daily GHG emissions were also found to be higher on weekends as time-use patterns shift from paid work to free-time activities, highlighting the need for mitigation strategies on a weekly scale.

**Supplementary Information:**

The online version contains supplementary material available at 10.1007/s11356-022-23387-w.

## Introduction

Global greenhouse gas (GHG) emissions have been rising in the past decades (Olivier et al. 2017), driven largely by meeting the energy demand of increasing household consumption (Druckman and Jackson 2016; Shigetomi et al. 2018; Wiedmann et al. 2020). Households are estimated to have contributed to over 70% of global GHG emissions through consumption (Hertwich and Peters 2009). Behavioral change has been found to be promising for reducing consumption-induced emissions (Dietz et al. 2009) and is increasingly regarded as important for the transition to more sustainable lifestyles (Schanes et al. 2016).

**Fig. 1:**
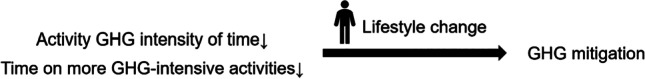
Carbon mitigation through behavioral change viewed from the time-use perspective

In existing studies, estimations of household consumption–induced GHG emissions and related discussions on emission mitigation through behavioral change have been approached overwhelmingly from a consumption-based perspective involving only monetary expenditures (Girod and de Haan 2009; Mi et al. 2019; Wiedenhofer et al. 2018). However, an closer examination of the daily household consumption behavior indicates that an actual time-use perspective combining concerns for both monetary and time budget should be adopted (Schipper 1989). Moreover, unlike monetary budgets, the available time of 24 h per day is universal and constant. This characteristic of time provides a novel perspective for constructing a more consistent and comprehensive framework for accounting household consumption behavior and exploring mitigation potential, such as the utility function (Becker 1965; Gronau 1977; Gronau and Hamermesh 2006), that contrasts with the traditional expenditure-based perspective. In the time-use perspective, we define GHG intensity of time, i.e., GHG emissions per unit time, of an activity. Total emissions from an activity are thus the product of its GHG intensity of time and activity time. As Fig. 1 suggests, carbon mitigation can thus be achieved through behavioral changes by households reducing the time spent on activities with higher GHG intensity of time, or changing the associated consumption structure that lowers the GHG intensity of time of activities.

Nevertheless, knowledge about the energy use and carbon footprint of household activities from a time-use perspective has been rare. Systematic estimation of household activity emissions has only been conducted in three existing studies focusing on the UK (Druckman et al. 2012), Austria (Smetschka et al. 2019), and China (Yu et al. 2019), respectively, while the estimation of activity energy intensity has been conducted only for the USA (Schipper 1989), the Netherlands (van der Werf 2002), Finland (Jalas 2002; Jalas and Juntunen 2015), and France (De Lauretis et al. 2017). Moreover, as Table 1 suggests, the methods adopted in these studies could be improved by increasing the coverage of total daily time, encompassing both direct and indirect energy use/emissions, or more importantly, adopting a finer disaggregation of daily time into activities which is expected to enhance the capacity of differentiating between similar activities and thus assist in identifying emission mitigation potential with a higher precision. Thus far, the previous study with the most detailed activity categorization recognizes as few as 20 daily activities (Smetschka et al. 2019). Comprehensive coverage of daily time and both indirect and direct emissions, along with detailed disaggregation of daily time into activities, should better differentiate between time-use patterns associated with daily household activities and therefore better capture the carbon footprint of household consumption behavior, which is fundamental for devising practical behavioral change strategies aiming at effective carbon mitigation. Furthermore, variations in weekly household time-use patterns, largely influenced by working time, could also lead to varying patterns of activity emissions during the week, which has not been discussed in existing studies. Instead of changing household time-use patterns on a daily basis, the carbon mitigation potential under the current work-life patterns might be inspected from the perspective of adopting particular behavioral changes based on the varied patterns of activities on weekdays and weekends.

**Table 1:** Overview of the few existing studies related to household time use and emissions/energy

Studies	Year	Country	Number of activity categories	Emission/energy coverage	Coverage of daily time
Schipper (1989)	1975, 1985	USA	4	Direct and the indirect energy use	100%
Jalas (2002)	1987	Finland	14	Direct and the indirect energy use	30%
Jalas (2005)	1987–1988, 1999–2000	Finland	15	Direct and the indirect energy use	78% (1987–1988), 80% (1999–2000)
Druckman et al. (2012)	2006	UK	18	Direct and indirect emissions	86%
Jalas and Juntunen (2015)	1987–1988, 1999–2000, 2009–2010	Finland	14	Direct energy and electricity	51%
De Lauretis et al. (2017)	2009–2010	France	15	Direct energy and electricity	100%
Smetschka et al. (2019)	2009–2010	Austria	20	Direct and indirect emissions	100%
Yu et al. (2019)	2008	China	14	Direct and indirect emissions	100%

As the third largest economy and a major global GHG emitter (Hirano et al. 2016; Jiang et al. 2020), Japan shows great promise for carbon mitigation (Kuriyama et al. 2019). Currently, no existing study has focused on the carbon footprint of Japanese household activities in detail and the related mitigation potential. This study therefore aims to bridge the aforementioned gaps by estimating the carbon footprint of Japanese households’ daily activities from a time-use perspective in detail. A detailed 85-activity disaggregation of household daily time enables us to differentiate between different time-use behaviors at a significantly improved level compared to previous studies. Meanwhile, weekly patterns in activity emissions are also estimated from weekly time-use patterns. An analysis of the carbon mitigation potential for Japanese households and a comparison with previous studies are made subsequently based on the findings.

The remainder of this paper is organized into three sections. The “Methods and materials” section presents a description of the methods and materials employed for the estimation of activity emissions and GHG intensity of time. The “Results” section presents the estimation results. The “Sensitivity analysis of the matching between NSFIE and STULA items” section presents a discussion of the results. The “Conclusion” section concludes the study.

## Methods and materials

**Fig. 2:**
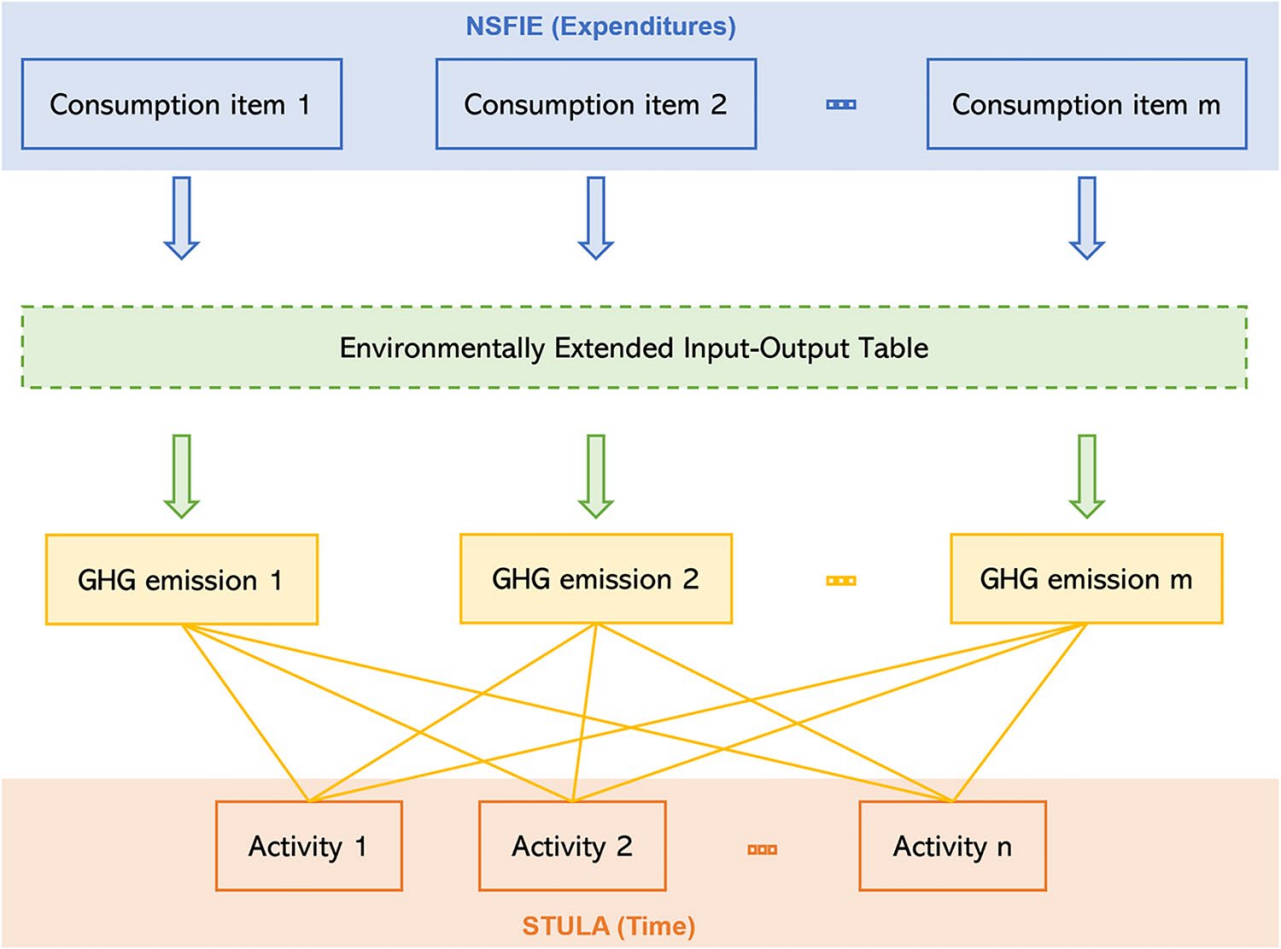
Methodological framework

In this study, we regard household lifestyles as being composed of single activities quantitatively represented by monetary expenditure and time length. Estimating the carbon footprint of household activities is a two-stage process. First, using data on household expenditures and data on the GHG intensity of expenditure derived from EEIOA, we calculate direct emissions and indirect emissions of household consumption items. Second, we allocate these direct and indirect emissions to various daily household activities at different shares reflecting the use of consumption items by different activities, to obtain the GHG emissions of each of the daily household activities. Figure 2 illustrates the abovementioned methodology. Details regarding the datasets and methods are provided in the following sections.

### Data on household expenditures and time use

Lifestyle-related data includes the microdata of the National Survey of Family Income and Expenditure (NSFIE) (2004) and the Survey of Time Use and Leisure Activities (STULA) (2006a), which are conducted by the Ministry of Internal Affairs and Communications (MIC) on a 5-year basis. The latest versions of the two surveys for which microdata is available are 2004 NSFIE and 2006 STULA. GHG emissions are estimated from household expenditures using data on the GHG intensity of expenditure, i.e., the quantity of GHG emissions corresponding to per unit expenditure, which is derived from the data provided by Embodied Energy and Emission Intensity Data for Japan Using Input-Output Tables (3EID) (Nansai et al. 2002) developed using the method of environmentally extended input-output analysis (EEIOA), and by applying the globally extended 3EID developed by Nansai et al. (2012).

The 2004 NSFIE gathered responses from 48,007 households across all 47 prefectures of Japan regarding their expenditures on a total of 320 consumption items belonging to 10 different categories from September to November 2004. The socioeconomic and demographic characteristics of households, such as household size, annual income, and age of household members, and the ownership of durable goods, including household appliances and cars, were also surveyed by the 2004 NSFIE.

The 2006 STULA contains two questionnaires — A and B. Both questionnaires surveyed members over 10 years old in households residing in all 47 prefectures of Japan about their activity patterns by time of the day at 15-min intervals on two consecutive days during the survey period but differed in sample size and the number of activity categories. Questionnaire A classifies daily behavior into 20 activities and garnered responses from a total of 351,202 respondents. In contrast, Questionnaire B classifies daily behavior into 85 activities with 18,291 records of time use from members of 3866 households. We adopted the microdata of Questionnaire B in this study as its 85-activity categorization constitutes a much more detailed description of daily behavior, although Questionnaire A draws upon larger sample size. The 85 activities can be aggregated into six activity categories, as shown in Table 2.

**Table 2:** Categorization of the 85 daily household activities of STULA

Category	Activity
Paid work	Main job; trips during main job; side job; trips during side job; commuting for work; rest during work; job hunting
Housekeeping	Management of meals; dessert-making; gardening; house maintenance; clothes maintenance; clothes-making; building and repairing; vehicle maintenance; household management; family care; family support; other housework; baby nursing; baby care; playing with babies; accompanying children; child education; accompanying children to and from school; shopping administrative services; commercial services; trips for housework; volunteering; trips for volunteering
Schoolwork, study, and research	Classes and other school activities; homework; private tutoring; school recess; commuting to school; study and research (extracurricular)
Personal care	Sleep; nap; medical treatment; medical examination; bathing; personal care; personal care (personal services); breakfast; lunch; dinner; late-night snack; light meals
Free time	Social activities; worship or sutra-chanting; ceremonial occasions; face-to-face socializing; familial communication; communication via telephone; communication via e-mail; communication via mail; entertainment and recreation; artistic creation; sweet-making (as hobby); productive; gardening (as hobby); pet care; walking the dog; clothes-making (as hobby); hobbies gaming; driving for pleasure; other hobbies; aerobic sports; ball games; water sports; productive sports; other sports; reading books; reading newspapers or magazines; watching TV; watching video and DVDs; listening to the radio; listening to recordings; resting
Other	Trips for hobbies; other trips; STULA-related activities; other activities

### Estimating the carbon footprint of consumption

The direct and indirect GHG emissions of a household consumption item are expressed as the product of the expenditure on the item and its emission intensity of expenditure:

1 $${E}_i^{di}={e}_i^{di}{Exp}_i$$

2 $${E}_i^{in}={e}_i^{in}{Exp}_i$$

where ${E}_i^{di}$ and ${E}_i^{in}$ correspond to the direct and indirect emissions of item *i*, and ${e}_i^{di}$ and ${e}_i^{in}$ represent the direct and indirect emission intensity of expenditure of item *i*, respectively, and *Exp*_*i*_ represents the household expenditure on item *i*. The total emissions from the consumption of item *i* are thus

3 $${E}_i^{tot}={E}_i^{di}+{E}_i^{in}$$

Although Eqs. (1) and (2) are identical in form, the direct emission intensity of expenditure and the indirect emission intensity of expenditure are derived separately. Direct emissions are GHG emitted directly in the process of households using purchased goods and services, while indirect emissions are GHG arising directly in other sectors. In addition to CO_2_ which results from the combustion of fossil fuels, e.g., city gas, kerosene, liquid propane, and gasoline, in this study, direct emissions also entail the other GHGs such as CH_4_, N_2_O, and fluorinated gases (HFCs, PFCs, SF_6_). The GHG emissions are converted into CO_2_-equivalent (CO_2_e) according to the global warming potential of each gas. Direct GHG intensity of expenditure is obtained by dividing the total GHG emissions provided in the 2005 3EID (Nansai et al. 2002) by the total household expenditures on items leading to direct emissions obtained by expanding the list of household expenditures in the 2004 NSFIE (MIC 2004) with household expenditures provided by the 2004 Family Income and Expenditure Survey (FIES) (MIC 2021).

The indirect emission intensity of expenditure is derived using globally-extended data of the 2005 3EID (Nansai et al. 2012), which was originally derived based on the Leontief Inverse matrix (I − A)^−1^, which is widely used in input-output analysis (Leontief 1986). The EEIO model builds upon this relationship and the environmental impacts resulting from final purchaser’s demand can be expressed as:

4 $$\textrm{X}={\left(\textrm{I}-\textrm{A}\right)}^{-1}\textrm{F}$$

where *X* is the vector of domestic production, *I* is the identity matrix, *A* is the input coefficient matrix, and *F* is the vector of final demand. The GHG emissions from domestic production, P, can be expressed as

5 $$\textrm{P}=\hat{\textrm{E}}\textrm{X}=\hat{\textrm{E}}{\left(\textrm{I}-\textrm{A}\right)}^{-1}\textrm{F}$$

where $\hat{\textrm{E}}$ is a diagonal matrix of the emission intensity for each sector. In the original 2005 3EID (Nansai et al. 2002), the environmental burdens of imports into Japan are assumed to be the same as if imported goods are produced domestically, whereas environmental burdens of products tend to vary from country to country (Hertwich and Peters 2009; Wiedmann et al. 2015). In other words, the carbon footprint of household consumption extends beyond national borders due to a globalized supply chain (Peters et al. 2011; Weber and Matthews 2008; Wiedmann et al. 2015). Therefore, in 2012, Nansai et al. (2012) made a global extension to the original 2005 3EID by incorporating the environmental burdens induced in the global supply chain into the data of Japan, using a global link input–output (GLIO) model. In our estimation we adopt the emission intensity of the GLIO model and $\hat{\textrm{E}}$ is therefore the global emission intensity of expenditure. An inventory of the emission intensity of expenditure is then made by matching the emission intensity per unit expenditure of products from various economic sectors in globally extended 2005 3EID with the expanded list of expenditures combining the 2004 NSFIE and the 2004 FIES , based on the method of Ihara et al. (2009) and Jiang et al. (2020). To match the price level of 2006 STULA (2006a), the emission intensity of expenditure is further converted into 2006 prices using the consumer price index (CPI) of Japan (MIC 2020).

### Estimating carbon footprint of household activities

#### Allocating emissions of consumption items to activities

The final procedure required to estimate activity emissions is the allocation of GHG emissions resulting from household consumption to daily household activities. A central issue for establishing linkage concerns the quantitative relationship between the use of consumption items and each household activity. In existing estimations of activity GHG emissions from a time-use perspective that adopts a more aggregate grouping of household activities, an item of expenditure is usually wholly allocated to only one category of activity. In this sense, the matching between expenditures and activities can be described as “multiple-to-one” as multiple items of expenditure are matched with only one category of activity. However, when the number of activity categories is as many as 85, i.e., the number of activity categories identified for Questionnaire B of 2006 STULA (MIC 2006a), a consumption item is much less likely to be involved in only one of all activities concerned. This is especially true for goods such as electricity, which is utilized in a variety of activities for maintaining the basic functioning of daily life. Many other items also tend to be consumed for more than one of the 85 activities^1^. Thus, it is necessary to break down each expenditure into multiple portions with each corresponding to one activity. The matching of each portion of an expenditure item with an activity is determined using a variety of statistics, as is done in Ihara et al. (2010). The correspondence between consumption items and activities was first done independently by some of the co-authors, who have different lifestyles, referring to the explanatory note of the Questionnaire B of STULA (MIC 2006b) and that of NSFIE (MIC 2004), and was then compiled into a single correspondence table after discussions. For nondurable and durable goods, the shares of each portion are determined according to the relative time length of all relevant activities. This allocation scheme is relatively reasonable for material consumption, which is usually positively related to the temporal length of an activity^2^. However, for electricity and water, the shares are based on a number of surveys regarding electricity use and water use (see Ihara et al. (2010)). Emissions from consumption items that are used by a specific activity are instead allocated to the corresponding activity alone. For goods and services that do not take up time for consumption, the allocation is based on the other time-consuming goods and services which make use of these goods and services. In addition to the abovementioned materials, the allocation also refers to the explanatory note of the Questionnaire B of STULA (MIC 2006b), which defines the scope of each activity and provides related examples. The matching between all expenditure items in NSFIE and all activities in STULA is provided in Table S1.

For a consumption item *ⅈ* and an activity *j*, the share *a*_*ij*_ of *ⅈ* being used for *j* is defined as

6 $${a}_{ij}=\frac{t_j}{\sum_j{t}_{ij}}$$

where *t*_*j*_ is the total length of time spent on activity *j*. *t*_*ij*_ is the time length of activity *j* that involves the consumption of consumption item *ⅈ*. $\sum_j{t}_{ij}$ is thus the total length of the time for all the activities that involve consumption item *ⅈ*. Equation (4) implies that *a*_*ij*_ should satisfy

7 $$\sum_j{a}_{ij}=1$$

after all activities, *j* that use consumption the item *ⅈ* have been accounted.

**Fig. 3:**
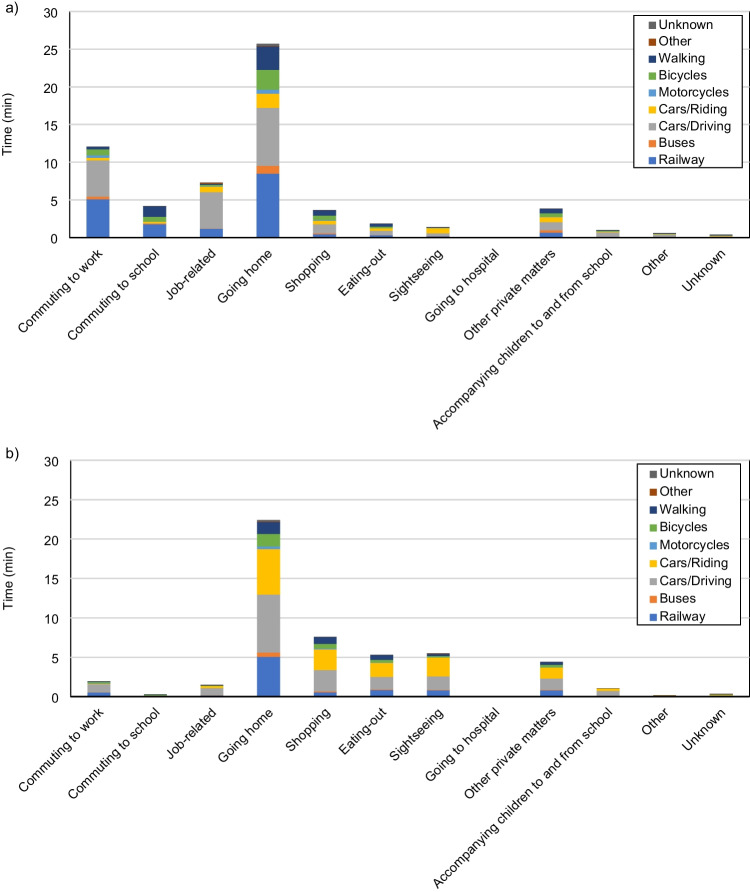
Use of transportation for different purposes **a** on weekdays and **b** on weekends, compiled from the data provided by NSUTC (Ministry of Land, Infrastructure, Transport and Tourism 2015)

For the allocation of household expenditures on transportation and energy to the corresponding activities, data from existing surveys on transportation and energy use are utilized to assist in the process. For expenditures on transportation, such as gasoline and traveling expenses, we assume that the shares of expenditure for related activities are proportional to the length of travel time for each of the activities. A similar assumption was adopted by Druckman et al. (2012) in their estimation of the GHG intensity of activities of British adults. To estimate the relative length of time for each means of transportation used in each activity, we used the results of the National Survey on Urban Transportation Characteristics (NSUTC) (Ministry of Land, Infrastructure, Transport and Tourism 2015) conducted by the Ministry of Land, Infrastructure, Transport and Tourism (MLIT). The 2005 NSUTC sampled household members above 5 years old from around 32,000 urban households in 62 cities of diverse sizes around Japan and collected information on the average number of trips by purpose and by means of transportation. Given that the average number of trips per day alone is not sufficient for establishing a relationship with travel time, and the 2005 NSUTC does not provide the average length of time per trip, we multiply the average number of trips per day surveyed by 2005 NSUTC with the average length of time per trip surveyed by the 2015 NSUTC, which produces the average length of time by means of transportation per day. Figure 3 shows the derived temporal lengths of different traveling purposes on weekdays and on weekends by means of transportation. The shares of transportation-related expenditure used for different transportation-related activities are calculated using Eq. (4), after matching the purposes of transportation in NSUTC with each of the activities.

**Fig. 4:**
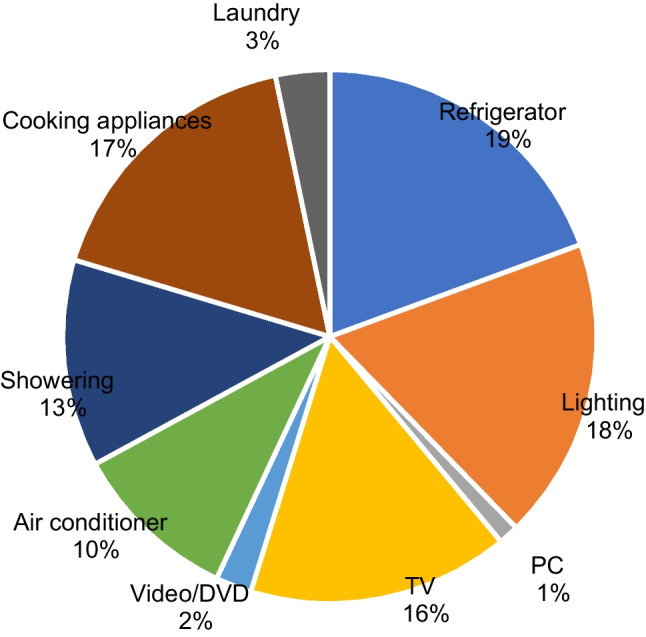
Share of household electricity use by purpose, compiled based on the 2009 Survey on Energy Consumption of the Household Sector (Ministry of Economy, Trade and Industry 2010)

As there is a lack of regular surveys on the average wattage and energy consumption by household appliances in Japan, the summary of the 2009 Survey on Energy Consumption of the Household Sector (Ministry of Economy, Trade and Industry 2010) conducted by the Agency for Natural Resources and Energy of the Ministry of Economy, Trade and Industry (METI) of Japan is utilized to derive the shares of energy-related expenditures in household activities. The survey reports on the shares of electricity use and the shares of total energy use by various household appliances. The relative power consumption by each of the household appliances is then derived by dividing the share of energy use by the total time of activities that make use of the appliance. In addition, considering that household use of personal computers (PC) in 2010 should have increased from 2006 at the cost of the time for watching TV, and the power consumption by a PC is around the same level as that of a TV set, the electricity used by PC and TV is integrated to estimate the shares of GHG emissions from electricity allocated to related activities. Figure 4 shows the share of household electricity use by purpose. It should be noted that the energy rebound effect (Sorrell 2007) should exist, which means that the savings due to improvements in energy efficiency would lead to more intensive use of household appliances, and including this effect could lead to more accurate estimation of the relative power consumption for households with different socioeconomic attributes. However, detailed data regarding the variations among the energy efficiency of appliances is lacking in Japan. The linkage of all 320 household expenditure items of the 2004 NSFIE and 85 time-use items of STULA is shown in Table S1.

In existing studies from the time-use perspective, some consumption items are excluded from the calculation of activity energy use/emissions owing to their involvement in more than one specific activity. Commonly excluded items are those regarded as “household infrastructure,” which are typically energy use by certain household goods and services such as heating and lighting, generally due to their diversity of use, and a lack of suitable criteria is generally the primary reason for excluding these items. However, not all existing studies exclude infrastructural items. For instance, in Druckman et al. (2012), heating and lighting are allocated to activities using time as a guiding factor, on the ground that related emissions can still be allocated to the activities even when household members are not present. Furthermore, it should be noted that not all relevant existing studies have excluded consumption items. For example, Smetschka et al. (2019), aiming at a comprehensive analysis from the functional time-use perspective, does not exclude any consumption items. Table 3 summarizes the excluded consumption items and the corresponding rationale for exclusion in each relevant existing study. Aiming at comprehensively capturing the landscape of household activity emissions, emissions from all consumption items are allocated to activities in this study for three reasons. First, including all expenditures provides a comprehensive and detailed picture of GHG emissions from household consumption behavior, while in existing studies, activity emissions do not add up to the total household consumption–induced emissions. Second, there lacks agreed criteria for selecting the range of expenditures to be included in the calculation, which impairs the consistency of estimates and applicability of methods. Third, as emissions from consumption items that are independent of time use are allocated to all activities proportional to activity time length, GHG intensity of time of different activities can be compared in both relative and absolute terms.

**Table 3:** Consumption items excluded in the relevant existing studies and the corresponding rationales

Studies	Year	Country	Excluded consumption items	Rationale
Schipper (1989)	1975, 1985	USA	Energy related to being at work and to vacations	Unspecified
Jalas (2002)	1987	Finland	Expenditures and energy uses that are related to household infrastructure; publicly provided goods such as the road infrastructure	Items constituting household infrastructure have a rather fixed demand and are connected to a variety of different activities
Jalas (2005)	1987–1988, 1999–2000	Finland	Goods with an infrastructure role such as housing and related heating and lighting; clothing in addition to housing-related expenditures	Many time-use categories are less-specifically defined by the objects that are being used; Due to the lack of suitable criteria or data for allocating energy requirements to specific activities
Druckman et al. (2012)	2006	UK	(COICOP categories)	Emissions due to furnishings, rent and financial services are excluded due to the difficulty in allocating; GHG emissions on holidays are excluded due to focus on time use during routine daily life; financial services, housing rental services, furnishings and textiles, postal services and tobacco use have been excluded as it is not possible to match any specific use of time to them.
			Tobacco and narcotics (2.2)	
			Rent paid for the housing (4.1)	
			Rent paid by owners occupying housing (4.2)	
			Furniture and furnishings, carpets and other floor	
			coverings (5.1)	
			Household textiles (5.2)	
			Postal services (8.1)	
			Package holidays (9.6)	
			Accommodation services (11.2)	
			Retirement homes, wet nurses, counsellors, adoption services etc. (12.4)	
			Insurance, financial and other services nec (12.5–12.7)	
			Holidays: Aviation and shipping emissions. Expenditure by UK residents abroad.	
Jalas and Juntunen (2015)	1987–1988, 1999–2000, 2009–2010	Finland	Heating related expenditures and energy use (43% of total energy use)	1) Buildings act as heat reservoirs and cause significant delay in regulating indoor temperatures; 2) few financial incentives for individuals to save and regulate heating due to no household specific measurement of heat and water consumption
De Lauretis et al. (2017)	2009–2010	France	Clothing; clothing accessories; tobacco products; insurance; all expenditures related to housing; all investments and taxes	Independence from the allocation of time
Smetschka et al. (2019)	2009–2010	Austria	None	None
Yu et al. (2019)	2008	China	A part of expenditures that cannot be allocated to specific activities	Difficulty in allocation

Some of the existing studies exclude a part of daily time, typically work time, due to their focuses on household emissions, which are not supposed to be allocated to this use of time, such as in Druckman et al. (2012) and Jalas (2002). Another possible reason for excluding work time by existing studies could be due to emissions arising from consumption during work time being recognized as within the boundary of the GHG inventory of companies stipulated by the corporate carbon accounting rules for Scopes 1, 2, and 3, which include the emissions arising from the activities of sources that are controlled or owned by companies, such as the heating of an office or producing cement by workers in a cement factory.

Nevertheless, this study finds it necessary to cover 100% of daily time for three reasons. First, as the carbon emissions of household activities are estimated from household expenditures, all concerned emissions in this study are due to household consumption and are thus not within Scopes 1 and 2, which respectively cover the direct and indirect GHG emissions occurring from sources that are controlled or owned by an organization (US Environmental Protection Agency n.d.). Moreover, emissions due to work-related trips such as business travel or employee commuting, which are Scope 3 emissions of companies, are not included in our calculation as these trips are generally subsidized by employers and are not part of household expenditures. Meanwhile, the emissions arising from household consumption of infrastructural items during work time, such as the clothes worn during working and the rent for housing, that are attributed to work-related activities in this study, are not covered by Scope 3 (World Resources Institute, World Business Council for Sustainable Development 2013) and therefore should not be attributed to companies. As such, the GHG emissions of work-related activities in this study, which arise from household consumption, are not in conflict with corporate carbon accounting rules. Second, although generally not much personal consumption is involved with working, work-related activities still compete with other activities for daily time, and changes in working time. As such, covering 100% of daily time enables treating time as an absolutely scarce resource, that is, increasing the time on one activity would definitely lead to decreases in the time spent on other activities. This would be beneficial for further studies that builds on the absolute scarcity and comprehensive inclusion of time, such as the assessment of time rebound effects, as is pointed out by Jalas (2002). Third, in reality, emissions from “infrastructural” household expenditures, such as clothes and rent, still occur during working time. Therefore, excluding work-related activities would lead to the exclusion of some consumption items, which contradicts the rationale for including all consumption items, i.e., comprehensiveness, consistency, and comparability, and would also fail to reflect the absolute values of activity emissions/GHG intensity of time. Among existing studies, Smetschka et al. (2019) similarly includes 100% of daily time for fulfilling the functional time-use perspective, which aims at achieving a comprehensive analysis.

#### Deriving the GHG emissions and GHG intensity of time of activities

As the 2004 NSFIE and 2006 STULA were conducted in different years, it is necessary to adjust the household expenditures in 2004 for 2006 to reflect the reality of household consumption patterns better. Though NSFIE is conducted only once every 5 years, the FIES (Ministry of Internal Affairs and Communications 2021), also conducted by MIC but on a much smaller sample than NSFIE, is conducted monthly and therefore provides a time series of monthly household expenditures applicable to the adjustment of expenditures in the 2004 NSFIE. Expenditures in the 2004 NSFIE were first matched with the items in the 2004 FIES to form an expanded list of household expenditures. Next, the set of ratios of the weighted average was derived for annual expenditure per capita on each expenditure item in the 2004 FIES to those in the 2006 FIES. Household expenditures in the 2004 NSFIE were then converted into the estimated expenditures in 2006 by multiplying them with the ratios. Apart from household expenditures, the emission intensity of expenditure, derived from the globally extended 3EID data in 2005 (Nansai et al. 2012), is also adjusted for 2006 values by using the CPIs of Japan (Ministry of Internal Affairs and Communications 2020) in 2005 and 2006.

Combining Eqs. (3) and (6), we can obtain the total activity emission ${E}_j^{tot}$ of an activity *j*:

8 $${E}_j^{tot}=\sum_i{a}_{ij}{E}_i^{tot}$$

The GHG intensity of time for each activity is then obtained by dividing the total emission of an activity ${E}_j^{tot}$ by the corresponding time *t*_*j*_ spent on the activity:

9 $${I}_j^{tot}=\frac{E_j^{tot}}{t_j}$$

#### Sensitivity analysis of the correspondences between NSFIE and STULA items

As is mentioned in the “Allocating emissions of consumption items to activities” section, the correspondences between the expenditure items of 2004 NSFIE and the time-use items of 2006 STULA are first performed independently by some of the co-authors of this paper, and then through discussions, merged into one correspondence table (Table S1a). To examine the robustness of this method, a sensitivity analysis is performed to explore the impacts of merging the different correspondence tables on the results by examining the differences between the results obtained using different correspondence tables. For the sensitivity analysis, two tables, Table S1b and Table S1c, were created to maximize the incorporation of the difference of values of some co-authors. A total of 43, or about 13%, of the 320 expenditure items were originally corresponded differently with activities in the two tables. Meanwhile, these different correspondences are involved in 77, or 91%, of the 85 daily activities. Table 4 lists the expenditures and activities for which the correspondences are different in the two correspondence tables. It can be seen that the expenditures where correspondences differ are mainly items with multiple uses, such as facial tissue and rolled toilet paper and men’s jackets, while for daily time use, free-time activities such as ceremonial occasions and face-to-face socializing are the activities for which correspondences are most likely to differ.

**Table 4:** Expenditures (in 2004 NSFIE) and activities (in 2006 STULA) with different correspondences in the two correspondence tables

2004 NSFIE		2006 STULA
Major category	Expenditure	Activity
Food	Bread	Light meals
	Fresh milk	
	Yogurt	
	Fresh fruits	
	Preserved fruits	
	Sugar	
	Mayonnaise and dressing sauce	
	Jam	
	Others	Baby care
Housing	Rents for dwelling	Bathing
	Rents for land	
	Fire insurance premium	
Furniture and household utensils	Beds	Medical treatment
	Quilts	
	Blankets	
	Other bedding	
	Facial tissue and rolled toilet paper	Management of meals, dessert-making, baby nursing, baby care, personal care, sweet-making (as hobby)
	Detergent, house and kitchen	Vehicle maintenance
	Dealing charges of large-sized discarded	
Clothes and footwear	Men’s jackets	Shopping, administrative services, commercial services, trips for housework, volunteering, trips for volunteering, social activities, worship or sutra-chanting, ceremonial occasions, face-to-face socializing, entertainment and recreation, walking the dog, driving for pleasure, other hobbies, trips for hobbies, other trips
	Men’s shoes	Main job, side job, rest during work, job hunting, shopping, administrative services, commercial services, volunteering, medical treatment, medical examination, personal care, personal care (personal services), breakfast, lunch, dinner, late-night snack, light meals, social activities, worship or sutra-chanting, ceremonial occasions, face-to-face socializing, entertainment and recreation, Artistic creation, entertainment with rewards , hobbies, driving for pleasure, other hobbies, aerobic sports, ball games, water sports, productive sports, other sports
	Women’s shoes	
	Canvas shoes	
Transportation and communication	Highway fares	Commuting to school
	Other public transportation	Trips during main job, trips during side job, commuting to work, accompanying children to and from school, trips for housework, trips for volunteering, commuting to school
	Automotive parts	Vehicle maintenance
	Articles related to private transportation	
	Automotive maintenance and repairs	
	Postage	Shopping, administrative services, commercial services
	Mobile telephone	Communication via mail
Education	Batteries	Management of meals, dessert-making, gardening, house maintenance, building and repairing, hobbies, gaming, listening to the radio
	Admission fees, movies, plays, cultural establishments, etc.	Ceremonial occasions, face-to-face socializing
	Admission fees, sports	
	Rental fees, sport facilities	
	Admission and playing fees, amusement park	
	Other admission and game fees	
	Membership dues	
Other expenditures	Cosmetics	Baby care
	Umbrellas	Building and repairing, vehicle maintenance
	Bags	Management of meals, dessert-making, gardening, house maintenance, clothes maintenance, clothes-making, building and repairing, vehicle maintenance, household management, family care, family support, other housework, baby nursing, baby care, playing with babies, accompanying children, child education, accompanying children to and from school, nap, personal care, personal care (personal services), breakfast, lunch, dinner, late-night snack, light meals, telephone conversation, communication via e-mail, communication via mail, entertainment and recreation, sweet-making (as hobby), entertainment with rewards , gardening (as hobby), pet care, walking the dog, clothes-making (as hobby), hobbies, gaming, reading books, reading newspapers or magazines, watching TV, watching video and DVDs, listening to the radio, listening to recordings, resting, STULA-related activities
	Wedding expenses	Face-to-face socializing
	Funeral expenses	
	Other ceremonials	

In the sensitivity analysis, the constant variables are the overlapping part of the two correspondence, while the changing variables are the part where the two correspondence tables differ. Deviations of the daily emissions and the GHG intensity of time of activities obtained using each correspondence table from the results obtained using the merged table, which is actually used for the calculation in this study, are evaluated. The absolute percentage error (APE) for each activity and the mean absolute percentage error (MAPE) of each activity category (6 in total) and of all activities are the two metrics for measuring the deviations and are mathematically defined as:

10 $$\textrm{APE}=\left|\frac{A_i-{F}_i}{A_i}\right|$$

11 $$\textrm{MAPE}=\frac{1}{85}\sum\nolimits_{\textrm{i}\ }\left|\frac{A_i-{F}_i}{A_i}\right|$$

where *i* represents each of the 85 daily household activities listed in Table 2, *A*_*i*_ represents the actual values of daily emissions or GHG intensity of time obtained using the merged correspondence table, and *F*_*i*_ represents the values obtained using each of the correspondence tables, respectively. The results and the corresponding comparisons are presented in the “Sensitivity analysis of the matching between NSFIE and STULA items” section.

### Deriving weekly patterns of activity carbon footprint

Weekly patterns in the GHG emissions of household activities were calculated based on weekly household time-use patterns. Information regarding the days of the week on which a time-use record is filled, provided in the microdata of Questionnaire B of 2006 STULA, is employed to compile the weekly time-use patterns. The average weekly time used on the six categories of activity listed in Table 2 was derived by averaging the time spent on each of the activities for each day of the week. Because data regarding the use of consumption items for activities on different days of the week are lacking, we assume that households use the same combination of consumption items for activities during the whole week. The average GHG intensity of time for each of the activities derived following the process specified in the “Deriving the GHG emissions and GHG intensity of time of activities” section was multiplied directly with the time used by activities for each day of the week to derive the total daily GHG emissions of activities. Additionally, because the NSUTC also distinguishes between weekdays and weekends and reports separately on the average number of trips and the average length of time for each trip by different means of transportation for different purposes, the NSUTC data is also utilized for the allocation of emissions to activities on weekdays and on weekends, thus allowing for a description of the weekly patterns of the differences in transportation-related GHG emissions.

## Results

### Daily GHG emissions and GHG intensity of time of household activities

**Fig. 5:**
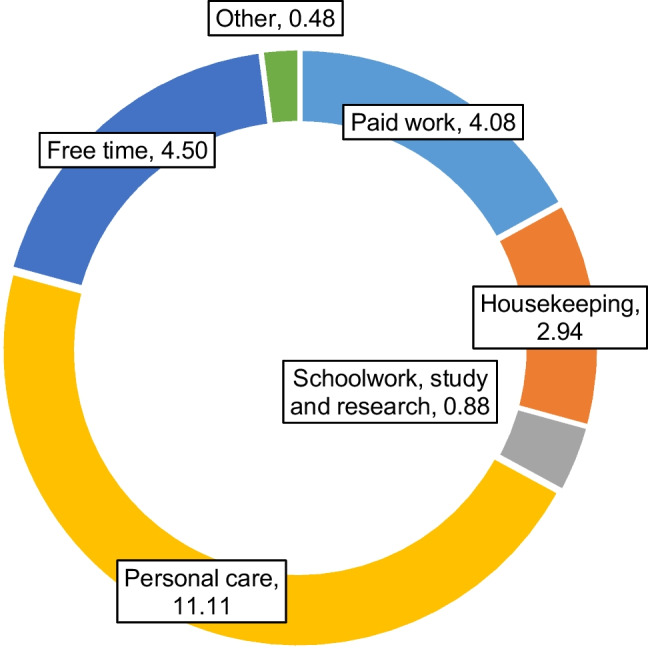
Per capita daily time (h) spent on 6 major activity categories by Japanese households

**Fig. 6:**
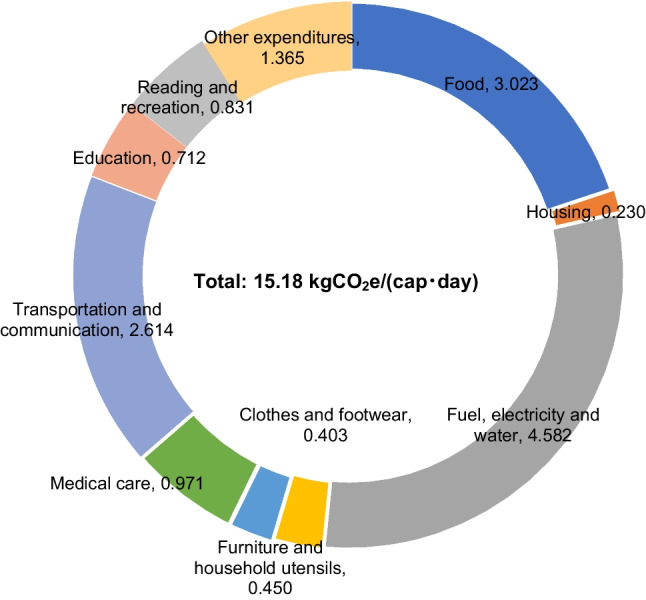
Per capita daily emissions by consumption category, in kgCO_2_e/(cap･day)

**Fig. 7:**
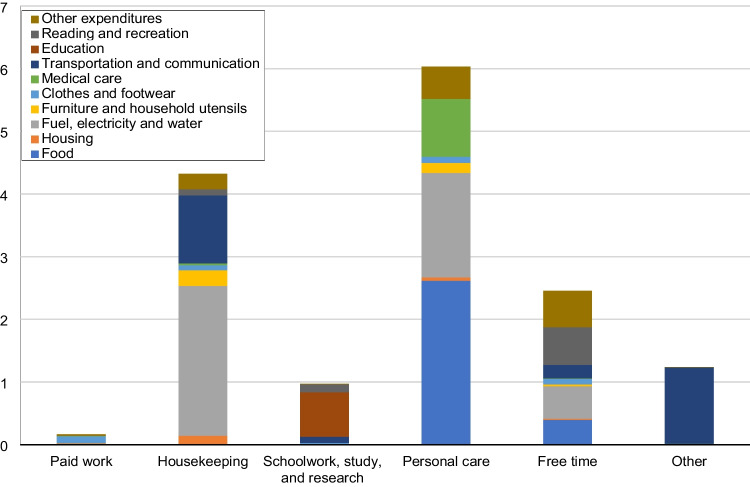
Per capita daily GHG emissions of the six major activity categories by consumption category

**Fig. 8:**
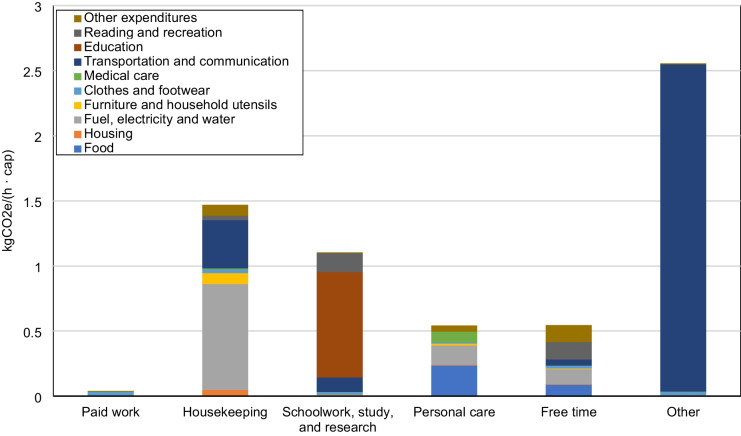
Average GHG intensity of time of the six major activity categories

The overall daily time-use patterns by residents in Japan in 2006 on the six categories of activities listed in Table 2 are shown in Fig. 5. More detailed information on the time spent on each of the 6 aggregated categories and the constituent 85 activities is listed in Appendix Table 7. Among the six activity categories, Personal care accounted for the largest share of the daily time budget of residents in Japan, occupying 11.11 h/(cap · day), with Sleep constituting much of the time. This is followed by activity categories Free time (4.50 h/(cap · day)), Paid work (4.08 h/(cap · day)), Housekeeping (2.94 h/(cap · day)), Schoolwork, study, and research (0.88 h/(cap · day)), and Other (0.48 h/(cap · day)). Total per capita daily GHG emissions induced by consumption are estimated to be 15.18 kgCO_2_e /(cap · day), or 0.63 kgCO_2_e/(h · cap). Fig. 6 shows the GHG emissions of the 10 major consumption categories. Table S2 lists the GHG emissions of all 320 expenditure items that constitute the 10 major consumption categories. Fig. 6 indicates that, among all 10 categories of household expenditures, expenditures on Fuel, electricity, and water, Food, and Transportation and communication are the biggest sources of GHG emissions relative to the other consumption categories. Activity categories with high GHG emissions are relatively more reliant on related consumption items, as suggested in Fig. 7, which shows the total per capita daily GHG emissions of the six activity categories by consumption category. Huge discrepancies exist between the GHG emissions of the activity categories. Calculating from household expenditures, the activity category with the highest average total GHG emissions from household consumption —Personal care — emits nearly 37 times as much as the activity with the lowest GHG emissions — Paid work, of which emissions are due to the consumption of infrastructural goods and services (e.g., clothes, housing rent) during work-related activities. Emissions of Personal care activities (6.03 kgCO_2_e/(cap · day)) are mostly contributed by the consumption of Food, Fuel, electricity and water, and Medical care. After personal care, GHG emissions from high to low are Housekeeping (4.32 kgCO_2_e/(cap · day)), Free time (2.46 kgCO_2_e/(cap · day)), Other (1.24 kgCO_2_e/(cap · day)), Schoolwork, study, and research (0.97 kgCO_2_e/(cap · day)), and Paid work (0.16 kgCO_2_e/(cap · day)). On the other hand, the average GHG intensity of time of each activity category, shown in Fig. 8, exhibits a different pattern from what is suggested by Fig. 7. The activity category with the highest average GHG intensity of time is Other, which amounts to 2.56 kgCO_2_e/(h · cap), contributed overwhelmingly by expenditures on Transportation and communication items used mainly for leisure trips. As Fig. 2 suggests, traveling purposes such as sightseeing (allocated to Other trips) have a high usage rate of cars (riding and driving), leading to high gasoline consumption and therefore higher GHG intensity of time than other means of transportation. At 1.47 kgCO_2_e/(h · cap), Housekeeping activities have the second highest GHG intensity of time, which is largely due to the consumption of Fuel, electricity, and water, mostly having direct emissions. This is followed by Schoolwork, study, and research (1.10 kgCO_2_e/(h · cap)), Free time (0.55 kgCO_2_e/(h · cap), Personal care (0.54 kgCO_2_e/(h · cap)), and Paid work (0.04 kgCO_2_e/(h · cap)). Paid work has the lowest GHG intensity of time and low total GHG emissions as a result of lower associated household expenditures, excluding Transportation and communication expenditures.

**Fig. 9:**
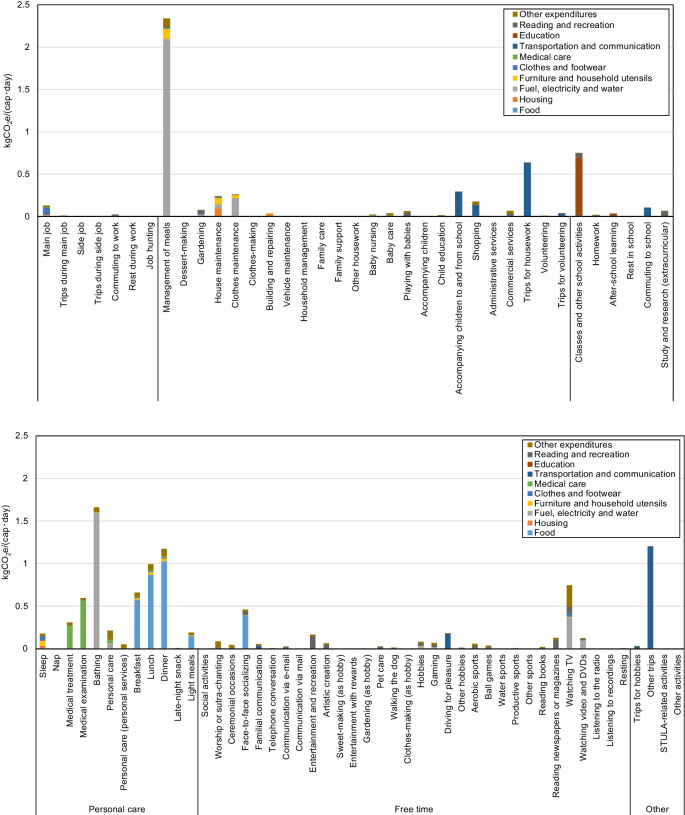
Average daily GHG emissions of 85 household activities categorized into 6 categories

**Fig. 10:**
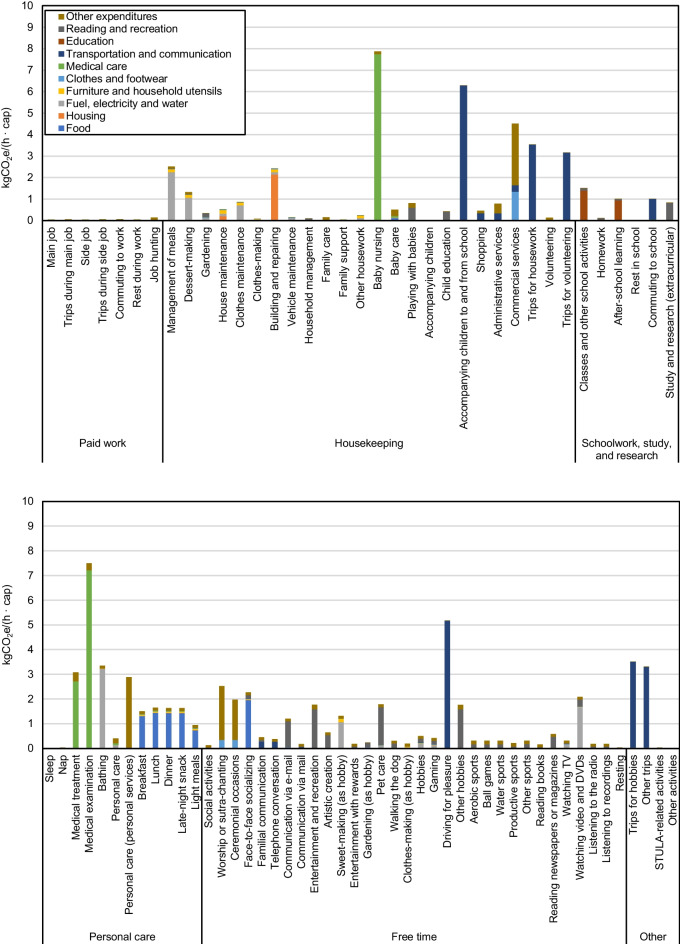
Average GHG intensity of time of 85 household activities categorized to 6 categories

A breakdown of the GHG emissions and GHG intensity of time of each activity category into various daily activities is shown in Figs. 9 and 10, respectively. Other activities, of the category Other, have no registered time use from respondents of the 2006 STULA. Its GHG emissions and GHG intensity of time are therefore set to zero. Figures 9 and 10 suggest significant discrepancies between the GHG emissions and the GHG intensity of time for activities within the same category. For Housekeeping activities, most GHG emissions are attributed to Management of meals (2.34 kgCO_2_e/(cap · day)), which includes preparations for meals such as cooking, dish washing, and pickle-making, whose emissions are mostly due to the consumption of Fuel, electricity, and water. Conversely, although having the highest GHG emissions among Housekeeping activities, the GHG intensity of time of Management of meals (2.52 kgCO_2_e/(h · cap)) lags behind Baby nursing (7.87 kgCO_2_e/(h · cap)), Accompanying children to and from school (6.29 kgCO_2_e/(h · cap)), and Commercial services (4.52 kgCO_2_e/(h · cap)) in the same category. Among activities related toSchoolwork, study, and research, Classes and Other school activities contribute most of the GHG emissions (0.75 kgCO_2_e/(cap · day)). For activities related to Personal care, Medical examination (0.60 kgCO_2_e/(cap · day)), Bathing (1.66 kgCO_2_e/(cap · day)), Breakfast (0.66 kgCO_2_e/(cap · day)), Lunch (0.99 kgCO_2_e/(cap · day)), and Dinner (1.17 kgCO_2_e/(cap · day)) account for most GHG emissions, which arise mainly from the consumption of goods and services of Medical care, Fuel, electricity, water, and Food. The highest GHG intensity of time occurs for Medical examination (7.50 kgCO_2_e/(h · cap)) in this activity category. Activities with heavy Medical care consumption, that is, Medical treatment, Baby nursing, and Medical examination, all have comparatively higher GHG intensity of time relative to other activities. Most Free time activities have relatively low GHG emissions and GHG intensity of time. Watching TV (0.75 kgCO_2_e/(cap · day)) and Face-to-face socializing (0.46 kgCO_2_e/(cap · day)) lead to relatively high GHG emissions in this category. Among the other activities, Other trips lead to 0.70 kgCO_2_e/(cap · day), corresponding to 4.01 kgCO_2_e/(h · cap). Transportation-related activities involving the heavier use of private cars generally have higher GHG intensity of time. Figure 10 suggests that Accompanying children to and from School (6.29 kgCO_2_e/(h · cap)), Driving for pleasure (5.18 kgCO_2_e/(h · cap)), Trips for housework (3.54 kgCO_2_e/(h · cap)), Trips for hobbies (3.52 kgCO_2_e/(h · cap)), and Other trips (3.31 kgCO_2_e/(h · cap)), which include mainly leisure trips, have relatively high GHG intensity of time compared to Commuting to school (1.00 kgCO_2_e/(h · cap)) — activities that rely more on less carbon-intensive means of transportation such as buses, trains, cycling, and walking.

**Fig. 11:**
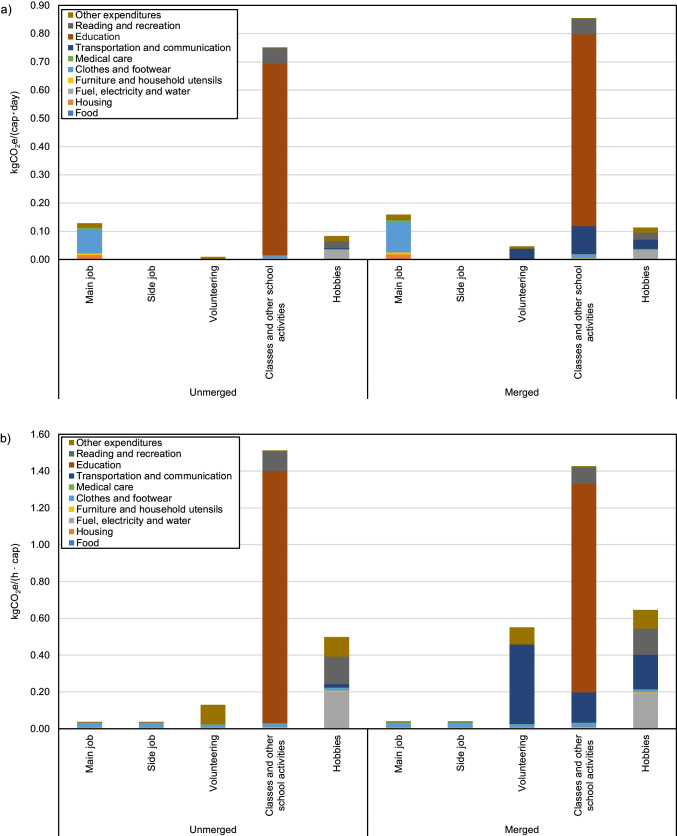
**a** Daily GHG emissions and **b** GHG intensity of time of six activities before and after merging with their corresponding trip activities

As trip activities are conducted to fulfill the purpose of their corresponding main activities, combining trips with their corresponding main activities could provide a new angle to examine the relative carbon intensity of household activities. Table 5 lists the 6 trip activities that are merged with their corresponding main activities. Other trip activities, including Trips for housework, Accompanying children to and from school, and Other trips, are not merged with main activities as they can be involved with multiple activities and the information on how much traveling is involved in each activity is unavailable. The emissions and time of commuting to work are distributed to Main job and Side job proportionally to the ratio of the emissions of trips during main job to the emissions of Trips during side job, which is about 99:1. Figure 11 shows a comparison between the GHG emissions and GHG intensity of time of these activities before and after the merging. On the one hand, the most notable increase in GHG emissions is for Volunteering (0.01 kgCO_2_e/(cap · day) to 0.05 kgCO_2_e/(cap · day)), by nearly 5 times, due to more frequent traveling for volunteering activities and the use of mainly privately-owned vehicles. By contrast, increases in the emissions of Classes and other school activities are much less significant (0.75 kgCO_2_e/(cap · day) to 0.80 kgCO_2_e/(cap · day)), due to the lower carbon intensity from Commuting to school. On the other hand, the GHG intensity of time of Volunteering soars from 0.13 kgCO_2_e/(h · cap) to 0.55 kgCO_2_e/(h · cap), and that of Hobbies soar from 0.50 kgCO_2_e/(h · cap) to 0.65 kgCO_2_e/(h · cap), while that of Classes and other school activities decreases from 1.51 kgCO_2_e/(h · cap) to 1.43 kgCO_2_e/(h · cap). For work-related activities, as the expenses are generally subsidized by employers in Japan, no significant changes are observed for GHG emissions and GHG intensity of time after merging with trip activities.

**Table 5:** Main activities and the merged trips

Main activities	Trips for main activities
Main job	Trips during main job, commuting to work
Side job	Trips during side job, commuting to work
Volunteering	Trips for volunteering
Classes and other school activities	Commuting to school
Hobbies	Trips for hobbies

### Weekly patterns of the daily GHG emissions of household activities

**Fig. 12:**
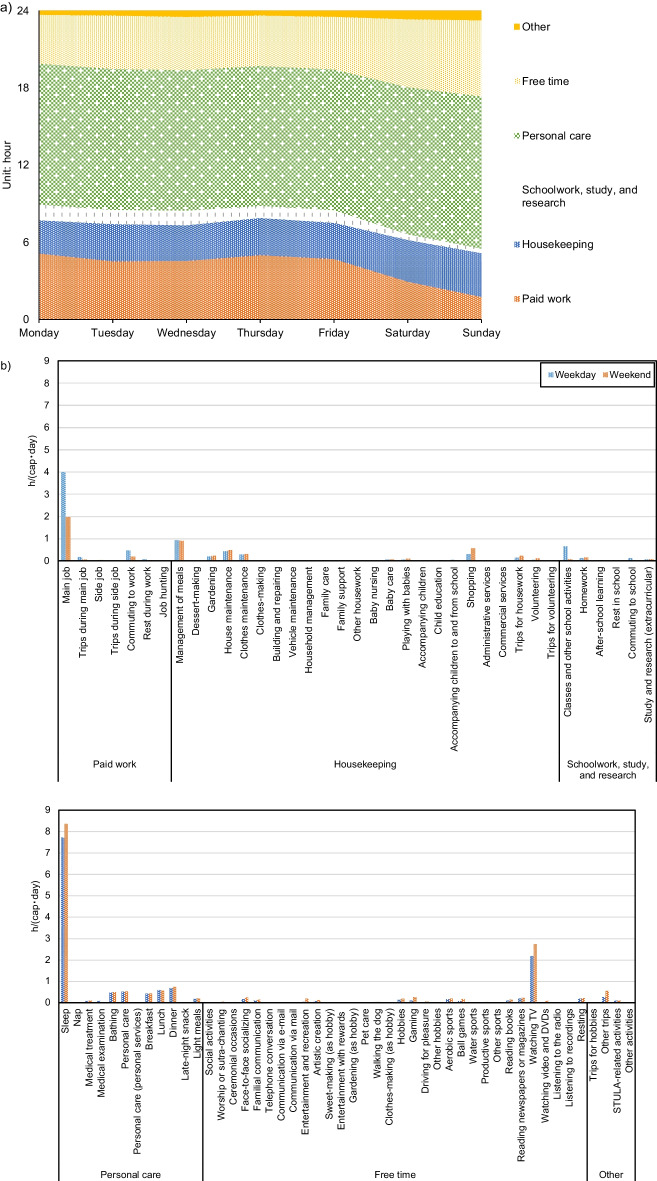
**a** Weekly time-use patterns by the 6 major categories of household activities and **b** time use on the 85 activities on weekdays and on the weekend in Japanese households in 2006

**Fig. 13:**
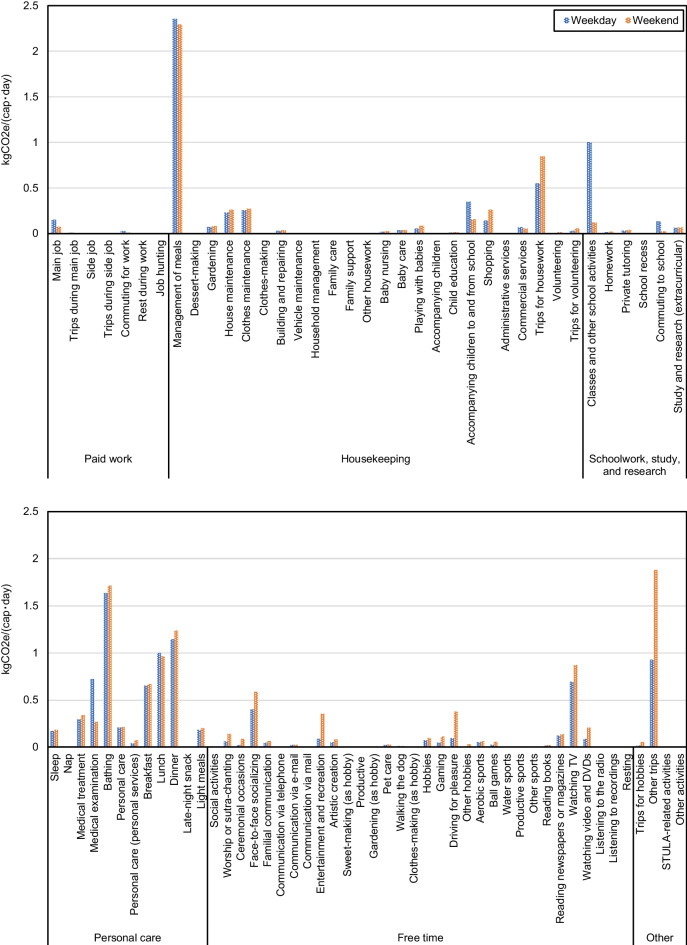
Per capita daily GHG emissions of 85 household activities on weekdays and on weekends

Questionnaire B of the 2006 STULA records the day of the week for each daily time record, which provides a convenient tool for us to look into the weekly patterns of daily household activities. We found that household time-use patterns vary among the different days of the week (Fig. 12). The most notable change is the trade-off in time used for Free time activities and Paid work, indicated by a decrease in the time for Paid work on weekends comparable with the increase in the time for Free time activities. The variability of time-use patterns within a week results in the discrepancies in activity emission patterns, as is shown in Fig. 13, assuming unvaried GHG intensity of time. The average daily per capita GHG emissions were estimated to be 14.77 kgCO_2_e/(cap · day) for weekdays and 16.20 kgCO_2_e/(cap · day) for weekends. The differences are admittedly not huge and are mainly due to an increase in the time spent on activities such as Free time activities, and Housekeeping, that is, activities with higher GHG intensity of time than work-related activities, and also due to a decrease in the time spent on activities of Schoolwork, study, and research, which have relatively high GHG intensity of time. At a more detailed level, Fig. 12 suggests that the average sleeping time increases from 7.7h on the weekdays to 8.4h on the weekend, which contributes to the low emission gap in emissions between the weekdays and the weekend.

Figure 13 shows the per capita GHG emissions of all activities on weekdays and on weekends, which provides us with insights into the major drivers of the variability in activity emissions within a week. The decrease in the GHG emissions of paid work is caused by a drop in the emissions from the three major emitter activities in this category. The decrease in emissions from Schoolwork, study, and research is similarly caused by a drop in the major emitter activity. The slight increase in emissions from Housekeeping activities is due to an increase in the emissions from activities such as Trips for housework, and Shopping outweighing the decrease in emissions of Management of meals, suggesting that on weekends, more time is allocated to housekeeping activities that occur outdoor. Most personal care activities see an increase in GHG emissions on weekends, whereas for medical examination, GHG emissions decrease significantly on weekends due to reduced time. An increase in GHG emissions can be seen in Fig. 13 for nearly all Free time activities and also for activities associated with traveling in the category other. The activity, Other trips, which includes mainly leisure trips such as family outings that are typically linked to gasoline use by private cars, has the biggest increase in GHG emissions among all activities in the two categories. This is the result of longer traveling time on weekends, indicating that long-distance trips are more likely to be conducted on weekends.

### Sensitivity analysis of the matching between NSFIE and STULA items

**Fig. 14:**
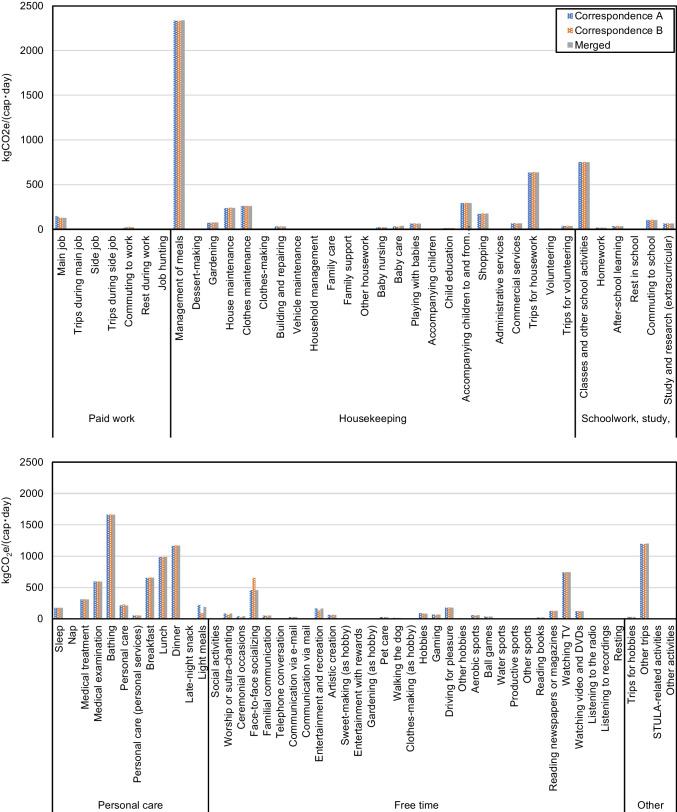
Daily GHG emissions of the 85 daily activities calculated using the two correspondence tables (A and B) and the merged correspondence table

**Fig. 15:**
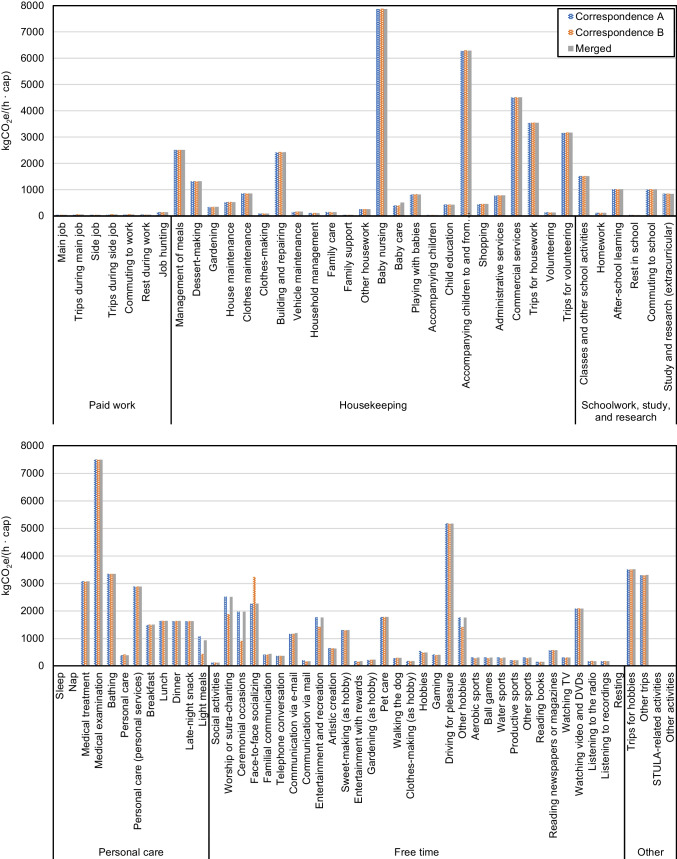
GHG intensity of time of the 85 daily activities calculated using the two correspondence tables (A and B) and the merged correspondence table

As is described in the “Sensitivity analysis of the correspondences between NSFIE and STULA items” section, a sensitivity analysis is performed to verify the robustness of the correspondence between 2004 NSFIE and STULA. The sensitivity analysis compares the outcomes obtained using two distinct correspondence tables (A and B, represented by Table S1b and Table S1c, respectively) with the outcomes obtained using the final merged table (Table S1a) to examine the extent to which results on activity emissions/GHG intensity of time are impacted. The daily emissions and the GHG intensity of time of the 85 daily activities calculated using the two correspondence tables (A and B) and the merged correspondence table (used for obtaining the results in this study) are shown in Figs. 14 and 15, respectively. For most activities, the daily GHG emissions and GHG intensity of time obtained from the three corresponding tables are similar, and Figs. 14 and 15 suggest that the differences are mostly likely to exist among Free time activities. Meanwhile, Table 6 lists the APEs and MAPEs for the daily emissions and GHG intensity of time of each activity. It can be seen that the overall MAPEs are below 0.05 for both correspondence A (0.033) and correspondence B (0.044), indicating that the impacts on the results obtained using the two corresponding tables due to their differences are minor, given that over 13% of the expenditure items corresponded differently with activities in the two tables. The most deviated activity category is Paid work, with the MAPE being 0.142 for correspondence table A. Nevertheless, as the emissions from Paid work are the least among the 6 activity categories and account or only 1% of daily emissions, the impacts on the overall consistency could be regarded as minor. Almost all MAPEs of the other 5 categories are below 0.05 for both correspondence tables A and B, some are even below 0.01, except for free-time activities calculated from correspondence table B, which at 0.072 is still regarded as moderate (below 0.01). The sensitivity analysis therefore suggests limited impacts on the consistency of results for the methods of corresponding 2004 NSFIE items and 2006 STULA items adopted in this study.

**Table 6:** APEs and MAPEs of the deviations of the daily emissions and GHG intensity of time obtained using each independent correspondence table from the results obtained using the merged table. The table does not discern between daily emissions and GHG intensity of time of activities as the APEs and MAPEs are same for both

Activities (2006 STULA)		Correspondence A		Correspondence B	
Major category	Activity	APE	MAPE	APE	MAPE
Paid work	Main job	0.121	0.142	0.006	0.062
	Trips during main job	0.209		0.140	
	Side job	0.121		0.006	
	Trips during side job	0.209		0.140	
	Commuting for work	0.203		0.136	
	Rest during work	0.101		0.005	
	Job hunting	0.033		0.002	
Housekeeping	Management of meals	0.002	0.026	0.003	0.013
	Dessert-making	0.005		0.006	
	Gardening	0.034		0.014	
	House maintenance	0.014		0.007	
	Clothes maintenance	0.002		0.000	
	Clothes-making	0.016		0.000	
	Building and repairing	0.006		0.002	
	Vehicle maintenance	0.091		0.013	
	Household management	0.013		0.000	
	Family care	0.009		0.000	
	Family support	0.035		0.000	
	Other housework	0.005		0.001	
	Baby nursing	0.000		0.001	
	Baby care	0.220		0.230	
	Playing with babies	0.007		0.007	
	Accompanying children	0.042		0.000	
	Child education	0.016		0.013	
	Taking children to and from school	0.002		0.001	
	Shopping	0.025		0.000	
	Administrative services	0.015		0.000	
	Commercial services	0.003		0.000	
	Trips for housework	0.002		0.002	
	Volunteering	0.055		0.000	
	Trips for volunteering	0.003		0.002	
Schoolwork, study, and research	Classes and other school activities	0.001	0.014	0.000	0.003
	Homework	0.013		0.000	
	Private tutoring	0.002		0.000	
	School recess	0.048		0.000	
	Going to school	0.011		0.008	
	Study and research (extracurricular)	0.009		0.007	
Personal care	Sleep	0.002	0.019	0.002	0.050
	Nap	0.045		0.000	
	Medical treatment	0.002		0.001	
	Medical examination	0.001		0.000	
	Bathing	0.001		0.000	
	Personal care	0.005		0.073	
	Personal care (personal services)	0.001		0.000	
	Breakfast	0.006		0.000	
	Lunch	0.005		0.000	
	Dinner	0.005		0.000	
	Late-night snack	0.005		0.000	
	Light meals	0.154		0.528	
Free time	Social activities	0.038	0.025	0.018	0.072
	Worship or sutra-chanting	0.002		0.251	
	Ceremonial occasions	0.002		0.535	
	Face-to-face socializing	0.002		0.426	
	Familial communication	0.062		0.065	
	Communication via telephone	0.004		0.000	
	Communication via e-mail	0.025		0.024	
	Communication via mail	0.243		0.033	
	Entertainment and recreation	0.001		0.196	
	Artistic creation	0.021		0.009	
	Sweet-making (as hobby)	0.001		0.005	
	Entertainment with rewards	0.011		0.125	
	Gardening (as hobby)	0.050		0.003	
	Pet care	0.001		0.000	
	Walking the dog	0.045		0.000	
	Clothes-making (as hobby)	0.007		0.000	
	Hobbies	0.111		0.003	
	Gaming	0.012		0.030	
	Driving for pleasure	0.001		0.000	
	Other hobbies	0.003		0.195	
	Aerobic sports	0.016		0.089	
	Ball games	0.016		0.089	
	Water sports	0.016		0.089	
	Productive sports	0.023		0.000	
	Other sports	0.016		0.088	
	Reading books	0.009		0.000	
	Reading newspapers or magazines	0.002		0.000	
	Watching TV	0.004		0.000	
	Watching video and DVDs	0.002		0.003	
	Listening to the radio	0.007		0.022	
	Listening to recordings	0.007		0.000	
	Resting	0.036		0.000	
Other	Trips for hobbies	0.003	0.017	0.005	0.004
	Other trips	0.003		0.006	
	STULA-related activities	0.045		0.000	
	Other activities	0.000		0.000	
Total	Total		0.033		0.044

## Discussion

### Comparing Japan with other countries

Currently, only three studies, focusing on the UK (Druckman et al. 2012), Austria (Smetschka et al. 2019), and China (Yu et al. 2019), respectively, have estimated the GHG emissions of household activities. The average GHG intensity of activities in Japan (0.63 kgCO_2_e/(h · cap)) is lower than that in the UK (1.2 kgCO_2_e/(h · cap)) and Austria (1.3 kgCO_2_e/(h · cap)), but higher than that of urban and rural China, where most activity time is below 0.4 kgCO_2_e/(h · cap). Our findings that eating activities^3^ have relatively high GHG intensity of time is similar to the UK, Austria, and urban China. Conversely, compared with Japan, the GHG intensity of time of entertainment and cultural activities is outstandingly high in Austria, and is similarly moderate in the UK, while that of hobbies and games and reading is similar to Japan in both countries. For reading, our results indicate higher GHG intensity of time for Reading newspapers or magazines (0.58 kgCO_2_e/(h · cap)) than Reading books (0.16 kgCO_2_e/(h · cap)), which is not reflected in previous studies that adopt less disaggregated activity categories. High GHG intensity of time for medical care-related activities is also found in the UK. Differences between the findings for these countries could result from multiple causes, such as the different GHG intensity of expenditures, different time use or expenditure patterns, different categorization of activities that lead to different activities being included as entertainment and cultural activities, or the differences in the methods employed in the studies for matching expenditures with activities.

### Mitigation potential and strategies from the time-use perspective

Our findings suggest that the discrepancies between the carbon footprint of daily household activities not only stem from the length of time invested by households, but also largely from the different compositions of consumption items for each activity, which directly leads to different GHG intensity of time. As such, from the time-use perspective, instead of focusing only on consumption, the exploration of carbon mitigation potential should consider the possibility of changes in both time use and consumption composition of household activities. It is also worth noting that the GHG intensity of time is only an indication of the average speed at which emissions occur for an activity. On the one hand, for some activities, especially those involving the consumption of energy goods such as city gas, gasoline, and electricity (belonging to the consumption categories of Fuel, electricity, and water, and transportation and communication), emissions are more likely to be proportional to the length of activity time, as these energies are usually consumed by household appliances steadily over a period of time. Reallocating the length of time on energy-intensive activities therefore directly induces carbon mitigation. On the other hand, the consumption of non-energy goods, such as food or clothes, tends to have a weaker link between the length of activity time. For such activities, emissions are more likely to occur sporadically or intermittently. Strategies targeting these activities should therefore focus on transforming the structures of consumption into ones that lower the overall consumption at its occurrence.

A typical case concerns the two activities that lead to the most GHG emissions — Management of meals and Bathing — both relying heavily on the use of gas or electricity (from Fuel, electricity, and water) for cooking and water heating. From the viewpoint of shortening the time spent on carbon-intensive activities, promoting cooking practices that require a shorter time of heating, such as changing from stewing to stir frying, should be able to directly lower the energy consumption and emissions effectively for the Management of meal. The time saved from cooking behavior change, for instance, could be allocated to eating activities for which lengthening the activity time is unlikely to translate into extra food consumption as people’s food intake is relatively stable. In fact, the Japanese government has been promoting slower eating habits, such as spending more chewing while eating (Ministry of Agriculture, Forestry and Fisheries n.d.). A slower eating rate is also associated with lower odds of metabolic syndromes such as obesity and high blood pressure (Nagahama et al. 2014). As Management of meals leads to an average 2.51 kgCO_2_e/(h · cap) and takes up 0.93h per day, a 50% reduction in the activity time can thus lead to the reduction of 1.17 kgCO_2_e per capita per day, or 7.69% of total daily carbon footprint. Shortening bathing time is expected to introduce similar effects. A 20% reduction in daily bathing time, or about 0.1h, could lead to a reduction of 0.33 kgCO_2_e/ per capita per day, or 2.2% of daily per capita carbon footprint. Although reinvesting the saved time in other activities could lead to the rebound of total emissions (time rebound effects, such as in Brenčič and Young (2009)) to some extent, overall carbon mitigation is still achievable as long as saved time is reallocated to activities with a lower GHG intensity of time, such as Sleeping (0.02 kgCO_2_e/(h · cap)) or Reading books (0.16 kgCO_2_e/(h · cap)). Naturally, different activities are expected to afford different flexibility in adjusting activity time. For example, the time spent on Driving for pleasure (2.84 kgCO_2_e/(h · cap)) or Artistic creation (0.64 kgCO_2_e/(h · cap)) can be adjusted more easily than Main job (0.04 kgCO_2_e/(h · cap)) or Class and other school activities (1.51 kgCO_2_e/(h · cap)). Nevertheless, free-time activities and work or school-related activities are generally subject to weekly patterns, which will be further discussed later.

From the viewpoint of lowering the GHG intensity of time by transforming the composition of consumption, adopting more energy-efficient cooking or water heating appliances equipped with innovative technologies, or simply by shifting from electricity to city gas as the fuel for heating in Japanese households, can lead to a lower GHG intensity of time^4^. Promoting cooking practices that rely less on strong heat, for example stir-frying over deep-frying, or choosing ingredients that require less heat to get cooked, can also reduce the energy demand and the consequential carbon footprint of the management of meals. Another example is lowering the GHG intensity of time for activities related to mobility, for which emissions are largely due to direct energy use from the combustion of fossil fuels (from transportation and communication). Utilization rates of different transportation means are a principal factor behind the discrepancies among the GHG intensity of time of the various transportation-related activities. A typical example is the GHG intensity of time of Commuting to school (1.00 kgCO_2_e/(h · cap)) being much lower than other personal trips, due to the school commuter’s low reliance on driving, as Fig. 3 suggests. If other non-work trip activities could have the same GHG intensity of time as commuting to school while keeping activity time unchanged, the per capita daily emissions could be reduced by 1.73 kgCO_2_e, or 11.4%. However, it should be noted that changes in the means of transportation are usually accompanied by changes in traveling time. Apart from the general findings of improved energy efficiency by switching from privately owned cars to buses, rail, cycling, and walking (Lipscy and Schipper 2013), or encouraging the choice of closer destinations for traveling and improved urban planning (Haselsteiner et al. 2015; Heinonen et al. 2013; Ivanova et al. 2018), the time-use perspective suggests that, on the occasion that changes in transportation lead to longer traveling time, the overall energy use/carbon footprint might be reduced as the available time decreases for other activities, thereby discouraging the associated energy/material consumption.

Meanwhile, it is important that mitigation strategies from the time-use perspective be implemented without impairing subjective well-being. As is discussed in Druckman and Gatersleben (2019), activities that “involve physical and mental activity (and challenge), social contact through which people can satisfy basic psychological needs, and contribute to personal growth” are associated with greater subjective wellbeing, implying leisure activities. However, currently, there lack comprehensive and systematic assessments of the dynamics between subjective well-being and activity emissions, and more rigorous future studies would be desirable for filling in this gap.

### Mitigation potential implied by weekly activity patterns

The weekly variations in household time use and the consequential carbon footprint are largely a result of work-holiday patterns, reflected in the shift of time from paid work on the weekdays to free-time activities on the weekend. As average daily working time has been decreasing in Japan (Ministry of Health, Labour and Welfare 2022), and paid work activities have relatively high GHG intensity of time compared to many free-time activities, our findings imply a decrease in future carbon footprint if the trend of decreasing working time in Japan will not be reversed. Some previous studies have also similarly indicated that reducing working time will lead to lower energy use and environmental pressure, partly due to the resultant reduction in income that prompts people to adopt more contained consumption and energy use behavior (Devetter and Rousseau 2011; King and van den Bergh 2017; Nässén and Larsson 2015). There are, however, other studies that indicate the relationship between average working hours and societal GHG emissions to be mixed (Fitzgerald et al. 2018; Shao and Shen 2017). The time-use rebound effect triggered by changes in working time is also a factor that may complicate the assessment of mitigation potential, which according to Buhl and Acosta (2016) could include both the redistribution of time and losses in income. In a macroeconomic sense, the consequential changes in societal productivity due to changes in working time could alter the input-output matrix and thus influence the GHG intensity of expenditures, leading to further changes in household carbon footprint. The impacts of shortening working time on GHG emissions should be further studied in a holistic manner by treating household activities as a dynamic, interactive system.

Because most of the time spent on paid work and schoolwork is likely to be reallocated to free-time activities and thus leads to a significant rise in emissions on the weekend (Fig. 13), emission mitigation is therefore more likely to be achieved by targeting free-time activities on the weekend. Long-distance driving, for example, may be discouraged by charging higher tolls on weekends. Promoting activities such as artistic creation and gardening that have a low GHG intensity of time should also reduce the carbon footprint for free time. As activities of schoolwork, study, and research have relatively low GHG emissions, such as study and research (extracurricular), promoting lifelong learning should also lead to carbon mitigation, while aligning with the education policies in Japan (Ministry of Education, Culture, Sports, Science and Technology n.d.). The diffusion of technological innovations such as smartphones and tablets that are substitutes of TV, PC, etc.^5^ and are more energy-efficient is also likely to have already resulted in significant emission mitigation on the weekend from the relevant activities.

## Conclusion

This study is conducted with the aim of providing a detailed account of the patterns in the carbon footprint of daily Japanese household activities constituting their consumption behavior from the understudied time-use perspective, and investigating the associated carbon mitigation potential. Both direct and indirect emissions are covered, and the whole 24-h daily time is disaggregated into 6 major categories encompassing a total of 85 activities, a number much higher than the most detailed existing study (20 categories) by Smetschka et al. (2019). The detailedness enabled us to discover the discrepancies between the GHG emissions and GHG intensity of time of daily activities, especially for the similar ones that were previously regarded as the same activity categories, such as activities belonging to the categories of personal care and free time. With the application of the time-use perspective to Japanese household carbon footprint and the improved detailedness of time disaggregation, this study is able to enhance the quantitative basis for carbon mitigation policymaking that targets household consumption behavior. Strategies are likely to effectively achieve carbon mitigation effect by promoting changes in people’s cooking and eating practices, traveling habits, or by encouraging the choices of less carbon-intensive free-time activities.

This study also extends the time scale for inspecting thevariability in the emissions of household activities from the daily level to the weekly level based on weekly time-use patterns. Weekly variations in activity emissions are found to exist for Japan, especially from weekdays to weekends, though not huge. The variations are mainly driven by the work-holiday time-use patterns, reflected in the shift of time spent on paid work to free time from the weekdays to the weekend, and partially by activities related to schoolwork, personal care, and housekeeping. The effects of carbon mitigation by shortening working hours nevertheless need further inspection, as findings of previous studies indicate.

Overall, this study contributes novel information on the carbon footprint and its intensity of time of Japanese household activities from the time-use perspectives that are useful for evidence-based policymaking targeting household consumption behavior. In addition to carbon mitigation effects, as time use patterns are a part of household lifestyles, changes in time use patterns should also impact other aspects such as welfare and quality of life, as is indicated by Reisch (2015). Future research should therefore explore the ways for achieving the alleviation of environmental impacts while simultaneously maintaining households’ well-being during the transition to sustainable lifestyles.

### Major improvements over existing studies

The most significant improvement of this study over the few existing ones is in the detailed disaggregation of daily time into activities. Our more detailed disaggregation achieves better detailedness by dividing a large activity category into multiple activities. For example, in Smetschka et al. (2019), which so far has the most detailed disaggregation of daily time (20 activities), using hot water or personal hygiene products, and eating, which encompasses activities such as bathing, eating breakfast, lunch, or dinner, are all allocated to “Personal care.” By contrast, our study distinguishes between these activities, and the results indicate that the GHG emissions and intensity of time for bathing are both higher than those of the several eating-related activities. Another example is the free-time activities. The use of TV, radio, DVD, etc. for entertainment is regarded as a single activity category in both Smetschka et al. (2019) and Druckman et al. (2012), whereas our results reveal that watching videos and DVDs has a much higher GHG intensity of time than simply watching TV, which is less carbon-intensive with respective to time than reading newspapers or magazines but more so than reading books. These newly discovered differences indicate that more effective carbon mitigation could be achieved by prioritizing strategies that target those activities with higher GHG intensity of time than similar ones. Overall, as the 85-category disaggregation of daily time adopted in our study better accounts for individual needs, more details on the discrepancies between the carbon footprint of activities were able to be disclosed, which facilitates the practical advice about behavioral change towards a more sustainable lifestyle.

### Limitations of the study and desiderata for future research

This study has several limitations. The first limitation concerns the unavailability of newer survey microdata, which limits us to the use of older surveys — the 2004 NSFIE and 2006 STULA. As is discussed in the “Mitigation potential and strategies from the time-use perspective” section, household time-use patterns and the resultant GHG emission patterns for some activities might have changed since the surveys were conducted. Sekar et al. (2018) have indicated changes in time-use patterns regarding information and communications technology-based activities in the USA. However, this limitation can be overcome when later versions of the microdata of NSFIE and STULA become available. The results of this study can also serve as a reference for future estimates of activity emissions based on new data to reveal the historical trend in GHG emissions of household activities in Japan. New data regarding the allocation of consumption items to activities for more categories of goods and services can also improve the accuracy of the estimates.

Another limitation stems from the limited details on households’ purchase of the same type of consumption items with different prices and the corresponding carbon intensity. For instance, a cheap skirt and an expensive skirt can differ in prices significantly while having similar carbon contents, leading to largely different GHG intensity of expenditure and consequently different evaluation of GHG emissions for the expenditure item “Skirt” in 2004 NSFIE. Nevertheless, neither 2004 NSFIE nor 2005 3EID discerns between the qualities/prices of the goods and services of the same type, which therefore fails to reflect the impacts of the potential quality effects when comparing household emission patterns. Similar issues are also faced by existing studies, such as in Koide et al. (2019). As such, future studies might overcome this limitation by surveying on the spending patterns of the same types of goods and services among different households, and developing more refined data on household expenditures and GHG intensity of expenditure for the comparison of different household emission patterns induced by distinct consumption patterns.

Moreover, this study also matched the items in the time-use survey (2006 STULA) with the items in the expenditure survey (2004 NSFIE) based on values of co-authors with several different lifestyles (single-headed households and households with children). However, the number of people asked about their values is limited. A large-scale survey on the use of consumption items in activities will contribute to creating more realistic matching in the future.

The assumption of constant GHG intensity of time in our calculation of weekly emission patterns due to the lack of data on weekly household expenditure patterns poses another limitation. For example, on weekdays, face-to-face socializing is more likely to be conducted at home or within the proximity to home, whereas on weekends more likely to be conducted in an environment away from home, the corresponding structure of consumption should therefore be different due to this variability. The limitations could be addressed when information on weekly household expenditure patterns is available.

It should also be noted that, as household expenditures only represent household consumption behavior, final consumption in other sectors is not necessarily included in our calculation, such as the emissions from collective government spending on services that are not meant for individual consumption. This is likely to be a factor behind the per capita GHG emissions due to final consumption by the household sector (5.5 tCO_2_e/(cap･day) by our estimation) appearing to be lower compared to previous estimates of overall societal per capita emissions (13.8 tCO_2_e/(cap･day), according to Hertwich and Peters (2009)). Here, we present a case study situated in Japan, one of the developed countries that have been the focus of most existing studies. We expect future studies to also focus on the lifestyle of households in developing countries that are projected to be major drivers of future growth in energy consumption and GHG emissions.

### Supplementary information

ESM 1 (DOCX 82 kb)

## Data Availability

Available upon reasonable request.

## Appendix

**Table 7:** Time use by each of the 85 daily activities and each activity category

Category	Activity	Daily time (h)	Total time by category (h)
Paid work	Main job	3.421	4.08
	Trips during main job	0.151	
	Side job	0.032	
	Trips during side job	0.002	
	Commuting for work	0.402	
	Rest during work	0.067	
	Job hunting	0.004	
Housekeeping	Management of meals	0.930	2.94
	Dessert-making	0.002	
	Gardening	0.218	
	House maintenance	0.456	
	Clothes maintenance	0.305	
	Clothes-making	0.021	
	Building and repairing	0.013	
	Vehicle maintenance	0.009	
	Household management	0.022	
	Family care	0.040	
	Family support	0.015	
	Other housework	0.001	
	Baby nursing	0.003	
	Baby care	0.075	
	Playing with babies	0.078	
	Accompanying children	0.006	
	Child education	0.028	
	Accompanying children to and from school	0.047	
	Shopping	0.390	
	Administrative services	0.006	
	Commercial services	0.015	
	Trips for housework	0.180	
	Volunteering	0.073	
	Trips for volunteering	0.012	
Schoolwork, study, and research	Classes and other school activities	0.496	0.88
	Homework	0.147	
	After-school learning	0.033	
	Rest in school	0.023	
	Commuting for school	0.103	
	Study and research (extracurricular)	0.077	
Personal care	Sleep	7.909	11.11
	Nap	0.014	
	Medical treatment	0.101	
	Medical examination	0.079	
	Bathing	0.496	
	Personal care	0.532	
	Personal care (personal services)	0.018	
	Breakfast	0.437	
	Lunch	0.602	
	Dinner	0.716	
	Late-night snack	0.005	
	Light meals	0.202	
Free time	Social activities	0.023	4.50
	Worship or sutra-chanting	0.034	
	Ceremonial occasions	0.023	
	Face-to-face socializing	0.202	
	Familial communication	0.121	
	Telephone conversation	0.020	
	Communication via e-mail	0.022	
	Communication via mail	0.003	
	Entertainment and recreation	0.094	
	Artistic creation	0.098	
	Sweet-making (as hobby)	0.001	
	Productive	0.005	
	Gardening (as hobby)	0.018	
	Pet care	0.016	
	Walking the dog	0.046	
	Clothes-making (as hobby)	0.014	
	Hobbies	0.167	
	Gaming	0.165	
	Driving for pleasure	0.035	
	Other hobbies	0.007	
	Aerobic sports	0.185	
	Ball games	0.118	
	Water sports	0.010	
	Productive sports	0.030	
	Other sports	0.016	
	Reading books	0.129	
	Reading newspapers or magazines	0.223	
	Watching TV	2.358	
	Watching video and DVDs	0.060	
	Listening to the radio	0.028	
	Listening to recordings	0.023	
	Resting	0.208	
Other	Trips for hobbies	0.009	0.48
	Other trips	0.363	
	STULA-related activities	0.112	
	Other activities	0.000	

## Footnotes

1 For instance, TV sets are usually used not only for watching TV programs but also for gaming and playing CDs and DVDs.

2 For example, the longer a person showers, the more water, gas, and electricity used for bathroom lighting are consumed.

3 In the context of this study, these activities are breakfast, lunch, dinner, late-night snack, and light meals.

4 In Japan, the emission coefficient of electricity (around 0.5 kgCO_2_/kWh, or 0.139 tCO_2_/GJ) is much higher than that of city gas (0.050 tCO_2_/GJ) (Ministry of the Environment 2020) due to the heavy reliance on fossil fuels for power generation (Ministry of Economy, Trade and Industry 2019).

5 Mobile devices have attained a higher penetration rate than PC in Japan (Ministry of Internal Affairs and Communications 2019).
