# Supplementary material for: Estimating the carbon footprint of household activities in Japan from the time-use perspective
Source: Environ Sci Pollut Res Int. 2022 Oct 26;30(9):22343–74. doi: 10.1007/s11356-022-23387-w (PMC9938832; doi:10.1007/s11356-022-23387-w)
Supplement: Supplementary file 1 — (DOCX 82 kb) [file 11356_2022_23387_MOESM1_ESM.docx]

# Supplementary material

Table S1 – Correspondence of household expenditure items of the 2004 NSFIE with time-use items of 2006 STULA. “1” indicates a match and “0” indicates no match; a) merged correspondence table that is used for the calculation; b) correspondence table A; c) correspondence table B.

Table S2 – Daily per capita GHG emissions (in gCO_2_e/cap) of all 320 expenditure items constituting the 10 major consumption categories.
